# Risk of immune‐related pneumonitis for PD1/PD‐L1 inhibitors: Systematic review and network meta‐analysis

**DOI:** 10.1002/cam4.2104

**Published:** 2019-04-05

**Authors:** Yafang Huang, Haiyu Fan, Ning Li, Juan Du

**Affiliations:** ^1^ School of General Practice and Continuing Education Capital Medical University Beijing China; ^2^ Center of Stroke Beijing Institute for Brain Disorders Capital Medical University Beijing China; ^3^ Department of Library Capital Medical University Beijing China

**Keywords:** immune‐related pneumonitis, network meta‐analysis, PD1 inhibitor, PD‐L1 inhibitor, systematic review

## Abstract

**Background:**

Immune‐related pneumonitis is a clinically relevant and potentially life‐threatening adverse event. We performed a systematic review and network meta‐analysis to compare the risk of immune‐related pneumonitis among different PD1/PD‐L1 inhibitor‐related therapeutic regimens.

**Methods:**

Randomized controlled trials with PD1/PD‐L1 inhibitors were identified through comprehensive searches of multiple databases. Both published and unpublished data were extracted. Bayesian NMA was performed using random‐effects models. All‐grade (Grade 1‐5) and high‐grade (Grade 3‐5) immune‐related pneumonitis were estimated using odds ratios (ORs).

**Results:**

A total of 25 studies involving 16 005 patients were included. Compared with chemotherapy, the ORs of immune‐related all‐grade and high‐grade pneumonitis were significant for nivolumab (all‐grade: OR = 6.29, 95% CrI: 2.67‐16.75; high‐grade: OR = 5.95, 95% CrI: 2.35‐17.29), pembrolizumab (all‐grade: OR = 5.78, 95% CrI: 2.79‐13.24; high‐grade: OR = 5.33, 95% CrI: 2.49‐12.97), and nivolumab plus ipilimumab therapy (all‐grade: OR = 14.82, 95% CrI: 5.48‐47.97; high‐grade: OR = 15.26, 95% CrI: 5.05‐55.52). Compared with nivolumab, nivolumab plus ipilimumab therapy was associated with an increased risk of all‐grade pneumonitis (OR = 2.34, 95% CrI: 1.07‐5.77). Nivolumab plus ipilimumab therapy had the highest risk of both all‐grade and high‐grade pneumonitis among PD1/PD‐L1 inhibitor‐related therapeutic regimens.

**Conclusions:**

This study demonstrates that compared with chemotherapy, PD‐1 inhibitor may result in a higher risk of immune‐related pneumonitis. Nivolumab plus ipilimumab therapy had the highest pneumonitis risk. These findings could be taken into account by the physicians in decision making.

## 1 INTRODUCTION

Programmed cell death 1 (PD‐1) and programmed cell death‐ligand 1 (PD‐L1) monoclonal antibodies have shown significant clinical activity and marked efficacy in the treatment of advanced cancers.^1^, ^2^, ^3^, ^4^, ^5^ Many PD‐1 and PD‐L1 monoclonal antibodies have already been approved by Food and Drug Administration (FDA), consider for example, avelumab, atezolizumab, durvalumab, nivolumab, and pembrolizumab.^3^, ^4^, ^5^, ^6^, ^7^ These regulatory approvals have resulted in a widespread prescribing of PD1/PD‐L1 inhibitors for patients with advanced cancer.^8^ However, PD1/PD‐L1 inhibitors could disrupt normal immune tolerance mechanisms and be associated with immune‐related adverse events.^9^ Many organ systems and normal tissue would be affected.^9^, ^10^ Immune‐related pneumonitis is one of clinical relevant and potentially life‐threatening adverse events.^10^, ^11^

Although previous data from randomized controlled trials (RCTs) have already shown that PD1/PD‐L1 inhibitor‐related therapeutic regimens are likely to increase the risk of immune‐related pneumonitis,^1^, ^2^, ^3^, ^7^, ^12^ results from these RCTs are not consistent. Traditional systematic reviews and meta‐analyses were conducted to estimate the safety profile of PD1/PD‐L1 inhibitor.^13^, ^14^, ^15^, ^16^ However, due to lacking head‐to‐head direct evidence, comparative pneumonitis risk among different PD1/PD‐L1 inhibitor‐related therapeutic regimens have never been systematically studied.

Structured evidence on pneumonitis risk of PD1/PD‐L1 inhibitor‐related therapeutic regimens would be necessary for physicians in making clinical decisions. In this study, we carried out a systematic review and network meta‐analysis (NMA) to compare the immune‐related pneumonitis risk among different types of PD1/PD‐L1 inhibitor‐related therapeutic regimens simultaneously for cancer patients.

## 2 MATERIALS AND METHODS

### 2.1 Study design

We conducted a systematic review with both pairwise meta‐analysis and Bayesian NMA. The study was carried out according to the Cochrane handbook for systematic reviews of interventions.^17^ We reported the study according to the Preferred Reporting Items for Systematic Reviews and Meta‐Analyses (PRISMA) guidelines.^18^, ^19^ The study was registered in PROSPERO international prospective register of systematic reviews (CRD42018099163).

### 2.2 Search strategy and selection criteria

We systematically searched PubMed, Embase, Cochrane Central Register of Controlled Trials and ClinicalTrials.gov to identify potentially eligible studies. The terms used for the search strategy included “neoplasm”, “cancer”, “atezolizumab”, “avelumab”, “durvalumab”, “nivolumab”, “pembrolizumab”. There was no restriction on language or year of publication. We manually checked reference lists of related review articles to identify additional studies. The final date for the database running searches was June 19th, 2018.

Eligible studies had to be RCTs and should include either anti‐PD‐1 or anti‐PD‐L1 monoclonal antibody (ie atezolizumab, avelumab, durvalumab, nivolumab, pembrolizumab), alone or in combination with other types of treatment, in the intervention or control group. We evaluated the rates of immune‐related pneumonitis reported. We excluded studies only in abstract form and studies of quality of life or cost effectiveness analyses.

### 2.3 Study selection and data extraction

Two independent investigators (HY and FH) selected the potentially eligible studies and extracted the data from all the eligible studies. The titles, abstracts, and full‐text records were evaluated sequentially. The following information was extracted: title, trial name, year, funding sources, line of treatment, blinding, age, sex, tumor type, length of follow up, types and dosage of drugs, number of patients in the treatment and control arms, number of patients with pneumonitis of all‐grade (grade 1‐5) and high‐grade (grade 3‐5) in the treatment and control arms. Both published and unpublished data were extracted. The unpublished data were extracted from ClinicalTrials.gov.

### 2.4 Quality assessment

Two authors (HY and FH) independently assessed the risk of bias of included studies based on the Cochrane Collaboration's tool.^17^ Disagreement was resolved by discussion with a third author (LN). Six domains were evaluated: sequence generation, allocation concealment, blinding, incomplete outcome data, selective reporting, and other sources of bias.

We used the Grading of Recommendations Assessment, Development and Evaluation system (GRADE) approach to rate the quality of evidence.^20^ There were 4 levels of quality of evidence: high, moderate, low, and very low. The quality of evidence for each outcome was based on the fundamental study design and additional methodological factors.^20^

### 2.5 Outcome measures

The primary outcome of interest was all‐grade (grade 1‐5) pneumonitis. Secondary outcome was high‐grade (grade 3‐5) pneumonitis based on the National Cancer Institute Common Terminology Criteria for Adverse Events version 4.0.

### 2.6 Statistical analysis

DerSimonian‐Laird random effects model was used to perform traditional pairwise meta‐analyses. Summary effect size was presented as odds ratio (OR) for binary outcomes along with corresponding 95% confidence intervals (CIs). A 2‐sided *P* value of less than 0.05 or 95% CIs excluding one was regarded as statistically significant. Heterogeneity among studies was assessed using Cochrane *Q* statistic and quantified with *I* ^*2*^ statistic. Values over 50% indicated substantial heterogeneity.^17^ Publication bias was examined using funnel plots.^21^

Bayesian NMA allowing for indirect comparisons was performed to evaluate the risk of pneumonitis using a Markov Chain Monte Carlo (MCMC) simulation technique. We estimated the posterior distribution of all parameters using vague priors. We updated the MCMC model with 100000 simulated draws after a burn‐ins of 10000 iterations and used a thinning interval of 10 for each chain. We tested the adequacy of burn‐in and convergence using the Brooks‐Gelman‐Rubin statistic.^22^ Relative effects of treatments were reported as OR for binary outcomes along with corresponding 95% credible intervals (CrIs). All analyses were performed under the random‐effects model since they generally showed more conservative estimated effects and better goodness of fit. We calculated the posterior mean of the residual deviance to determine goodness of fit of the models. Ideally, each data point should contribute about one to the posterior mean of the residual deviance. Therefore, it can be compared with the number of data points for model fit checking. We used the loop‐specific approach to evaluate the presence of inconsistency.^23^ We calculated the values of 2 odds ratios (RoR) from direct and indirect evidence in the loop with 95% CI to assess the presence of inconsistency in each loop. Inconsistency was defined as disagreement between direct and indirect evidence with a 95% CI excluding zero.

Meta‐regression analyses were performed by adding prespecified covariates (ie median age, percentage of male, line of treatment, study phase, whether double‐blind was used) to the network meta‐analysis models. Sensitivity analyses were performed to evaluate the robustness of results based on the events reported in published articles only. The data analyses were conducted using STATA version 14.0 and WinBUGs version 1.4.3.

## 3 RESULTS

### 3.1 Eligible studies and patient characteristics

**Figure 1:**
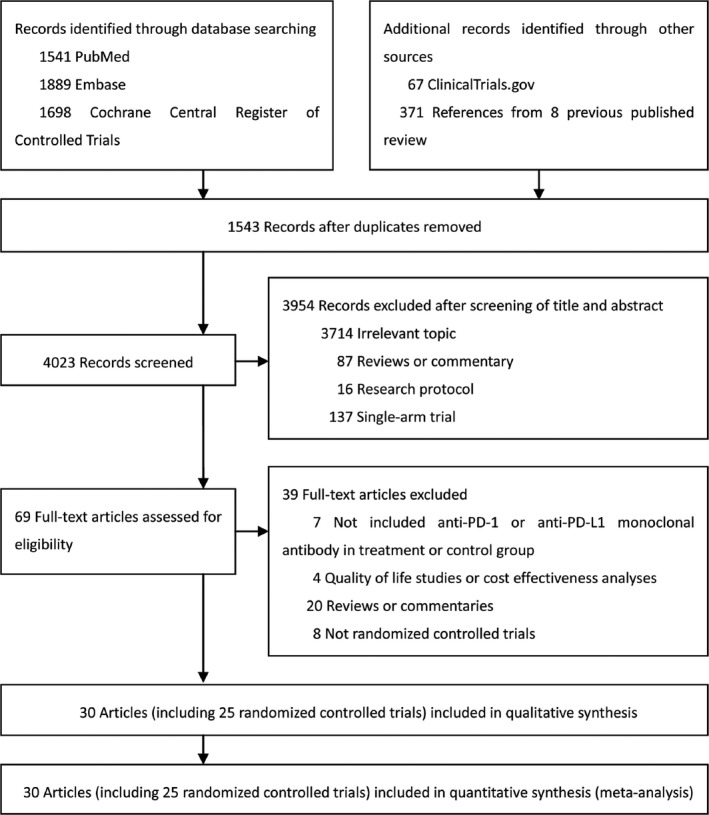
Literature search and selection

**Figure 2:**
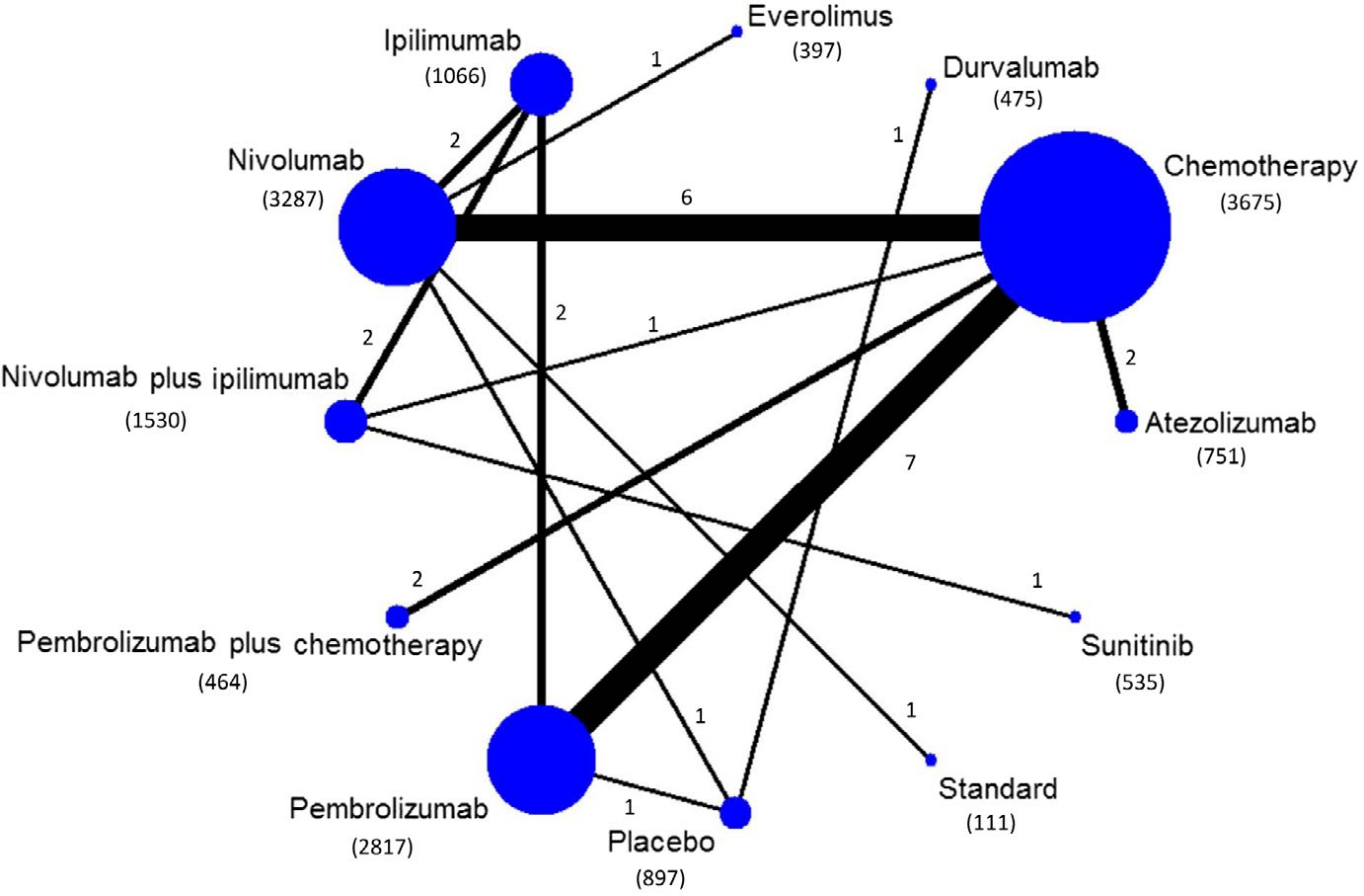
Network of eligible comparisons for the Bayesian network meta‐analysis. The size of the nodes is proportional to the number of patients (in parentheses) randomized to receive the treatment. The width of the lines is proportional to the number of comparisons (beside the line) comparing the connected treatment (nodes). A total of 25 randomized controlled trials included 30 comparisons were analyzed

Initial search identified 5566 records. 4023 potentially eligible articles were retrieved for detailed assessment. Thirty articles reporting 25 studies were included for meta‐analysis (Figure 1, Table S1). The 25 studies covered 12 types of treatment and involved a total of 16 005 cancer patients (Figure 2). The baseline characteristics of the included studies are listed in Tables 1 and 2 and Table S2. The risk of bias summary for included studies is listed in Table S3.

**Table 1:** Characteristics of eligible studies

Trial name	Year	Funding sources	Line of treatment	Study phase	Blinding	Median age	Age range	Sex (Male)	Tumor type	Length of follow up (month)	Treatment		
											Arm 1	Arm 2	Arm 3
CheckMate 017	2015	Bristol‐Myers Squibb	Second‐line	Phase 3	Open‐label	63	39‐85	208	Non–small‐cell lung cancer	Minimum 11	Nivolumab 3 mg/kg every 2 weeks	NA	Chemotherapy control
CheckMate 025	2015	Bristol‐Myers Squibb	Not clear	Phase 3	Open‐label	62	18‐88	619	Renal‐cell carcinoma	Minimum 14	Nivolumab 3 mg/kg every 2 weeks	NA	Everolimus 10 mg orally daily
CheckMate 026	2017	Bristol‐Myers Squibb	First‐line	Phase 3	Open‐label	64	29‐89	332	Non–small‐cell lung cancer	NA	Nivolumab 3 mg/kg every 2 weeks	NA	Chemotherapy control
CheckMate 037	2018;2015	Bristol‐Myers Squibb	Second‐line	Phase 3	Open‐label	60	23‐85	261	Melanoma	Median 8.4	Nivolumab 3 mg/kg every 2 weeks	NA	Chemotherapy control
CheckMate 057	2015	Bristol‐Myers Squibb	Second‐line	Phase 3	Open‐label	62	21‐85	319	Non–small‐cell lung cancer	Minimum 13.2	Nivolumab 3 mg/kg every 2 weeks	NA	Chemotherapy control
CheckMate 066	2015	Bristol‐Myers Squibb	First‐line	Phase 3	Double‐blind	65	18‐87	246	Melanoma	Median 8.9 and 6.8	Nivolumab 3 mg/kg every 2 weeks	NA	Chemotherapy control
CheckMate 067	2015;2017	Bristol‐Myers Squibb	First‐line	Phase 3	Double‐blind	60	18‐90	610	Melanoma	Median 12	Nivolumab 3 mg/kg every 2 weeks	Nivolumab 1 mg/kg every 3 weeks plus ipilimumab 3 mg/kg every 3 weeks	Ipilimumab 3 mg/kg every 3 weeks
CheckMate 069	2015	Bristol‐Myers Squibb	First‐line	Phase 2	Double‐blind	65	27‐87	95	Melanoma	Minimum 11	Nivolumab 1 mg/kg plus ipilimumab 3 mg/kg every 3 weeks	NA	Ipilimumab 3 mg/kg every 3 weeks
CheckMate 141	2016, 2018	Bristol‐Myers Squibb	Second‐line or more	Phase 3	Open‐label	60	28‐83	300	Head and neck carcinoma	Median 5.1	Nivolumab 3 mg/kg every 2 weeks	NA	Standard therapy control
CheckMate 214	2018	Bristol‐Myers Squibb	First‐line	Phase 3	Open‐label	62	21‐85	808	Renal‐cell carcinoma	Median 25.2	Nivolumab 3 mg/kg plus ipilimumab 1 mg/kg every 3 weeks, followed by nivolumab 3 mg/kg every 2 weeks	NA	Sunitinib 50 mg once daily for 4 weeks
CheckMate 227	2018	Bristol‐Myers Squibb	First‐line	Phase 3	Open‐label	64	29‐87	NA	Non–small‐cell lung cancer	Minimum 11.2	Nivolumab 3 mg/kg every 2 weeks plus ipilimumab 1 mg/kg every 6 weeks	Nivolumab 240 mg every 2 weeks	Chemotherapy control
CheckMate 238	2017	Bristol‐Myers Squibb and Ono Pharmaceutical	Not clear	Phase 3	Double‐blind	55	18‐86	527	Melanoma	Minimum 18	Nivolumab 3 mg/kg every 2 weeks	NA	Ipilimumab 10 mg/kg every 3 weeks for 4 doses and then every 12 weeks
KEYNOTE‐002	2015, 2017	Merck Sharp & Dohme, a subsidiary of Merck & Co.	Second‐line or more	Phase 2	Open‐label	62	15‐89	327	Melanoma	Median 10	Pembrolizumab 2 mg/kg every 3 weeks	Pembrolizumab 10 mg/kg every 3 weeks	Chemotherapy control
KEYNOTE‐006	2015;2017	Merck Sharp & Dohme, a subsidiary of Merck & Co.	First‐line or second‐line	Phase 3	Open‐label	62	18‐89	497	Melanoma	Median 7.9	Pembrolizumab 10 mg/kg every 2 weeks	Pembrolizumab 10 mg/kg every 3 weeks	Ipilimumab 3 mg/kg every 3 weeks
KEYNOTE‐010	2016	Merck & Co.	Second‐line or more	Phase 2/3	Open‐label	63	56‐69	634	Non–small‐cell lung cancer	Median 13.1	Pembrolizumab 2 mg/kg every 3 weeks	Pembrolizumab 10 mg/kg every 3 weeks	Chemotherapy control
KEYNOTE‐021	2016	Merck & Co.	First‐line	Phase 3	Open‐label	63	54‐70	48	Non–small‐cell lung cancer	Median 10.6	Pembrolizumab 200 mg every 3 weeks plus chemotherapy	NA	Chemotherapy control
KEYNOTE‐024	2016	Merck & Co.	First‐line	Phase 3	Open‐label	65	33‐90	187	Non–small‐cell lung cancer	Median 11.2	Pembrolizumab 200 mg every 3 weeks	NA	Chemotherapy control
KEYNOTE‐045	2017	Merck & Co.	Second‐line	Phase 3	Open‐label	66	26‐88	402	Urothelial carcinoma	Median 14.1	Pembrolizumab 200 mg every 3 weeks	NA	Chemotherapy control
KEYNOTE‐054	2018	Merck & Co.	Second‐line or more	Phase 3	Double‐blind	54	19‐88	628	Melanoma	Median 15	Pembrolizumab 200 mg every 3 weeks	NA	Placebo
KEYNOTE‐061	2018	Merck Sharp & Dohme, a subsidiary of Merck & Co.	First‐line	Phase 3	Open‐label	61	53‐70	410	Gastric or gastro‐oesophageal junction cancer	Median 7.9	Pembrolizumab 200 mg every 3 weeks	NA	Chemotherapy control
KEYNOTE‐189	2018	Merck & Co.	First‐line	Phase 3	Double‐blind	64	34‐84	363	Non–small‐cell lung cancer	Median 10.5	Pembrolizumab 200 mg every 3 weeks plus chemotherapy	NA	Chemotherapy control
OAK	2017	F. Hoffmann‐La Roche Ltd, Genentech, Inc.	Second‐line or more	Phase 3	Open‐label	63	33‐85	747	Non–small‐cell lung cancer	Minimum 19	Atezolizumab 1200 mg every 3 weeks	NA	Chemotherapy control
ONO‐4538‐12, ATTRACTION‐2	2017	Ono Pharmaceutical and Bristol‐Myers Squibb	Second‐line or more	Phase 3	Double‐blind	62	53‐69	348	Gastric or gastro‐oesophageal junction cancer	Median 12	Nivolumab 3 mg/kg every 2 weeks	NA	Placebo
PACIFIC study	2017	AstraZeneca	Second‐line or more	Phase 3	Double‐blind	64	23‐90	500	Non–small‐cell lung cancer	Median 14.5	Durvalumab 10 mg/kg every 2 weeks	NA	Placebo
POPLAR Study	2016	F. Hoffmann‐La Roche Ltd, Genentech, Inc.	Second‐line or more	Phase 2	Open‐label	62	36‐84	169	Non–small‐cell lung cancer	Median 13	Atezolizumab 1200 mg every 3 weeks	NA	Chemotherapy control

NA, not available.

**Table 2:** Number of patients with immune‐related pneumonitis (data for main analysis)

Trial name	Types of treatment			Number of patients for adverse events			Pneumonitis events (Grade 1‐5)			Pneumonitis events (Grade 3 ‐5)			Data source
	Arm 1	Arm 2	Arm 3	Arm 1	Arm 2	Arm 3	Arm 1	Arm 2	Arm 3	Arm 1	Arm 2	Arm 3	
CheckMate 017	Nivolumab	NA	Chemotherapy	131	NA	129	2	NA	0	2	NA	0	ClinicalTrials.gov
CheckMate 025	Nivolumab	NA	Everolimus	406	NA	397	25	NA	67	8	NA	12	ClinicalTrials.gov
CheckMate 026	Nivolumab	NA	Chemotherapy	267	NA	263	7	NA	0	7	NA	0	ClinicalTrials.gov
CheckMate 037	Nivolumab	NA	Chemotherapy	268	NA	102	1	NA	0	1	NA	0	ClinicalTrials.gov
CheckMate 057	Nivolumab	NA	Chemotherapy	287	NA	268	4	NA	0	4	NA	0	ClinicalTrials.gov
CheckMate 066	Nivolumab	NA	Chemotherapy	206	NA	205	2	NA	0	2	NA	0	ClinicalTrials.gov
CheckMate 067	Nivolumab	Nivolumab plus ipilimumab	Ipilimumab	313	313	311	2	6	2	2	6	2	ClinicalTrials.gov
CheckMate 069	Nivolumab plus ipilimumab	NA	Ipilimumab	94	NA	46	5	NA	0	5	NA	0	ClinicalTrials.gov
CheckMate 141	Nivolumab	NA	Standard therapy	236	NA	111	2	NA	0	2	NA	0	ClinicalTrials.gov
CheckMate 214	Nivolumab plus ipilimumab	NA	Sunitinib	547	NA	535	1	NA	0	1	NA	0	Published article
CheckMate 227	Nivolumab plus ipilimumab	Nivolumab	Chemotherapy	576	391	570	22	9	3	13	6	2	Published article
CheckMate 238	Nivolumab	NA	Ipilimumab	452	NA	453	6	NA	11	0	NA	4	Published article
KEYNOTE‐002	Pembrolizumab	Pembrolizumab	Chemotherapy	178	179	171	1	3	0	1	3	0	ClinicalTrials.gov
KEYNOTE‐006	Pembrolizumab	Pembrolizumab	Ipilimumab	278	277	256	2	2	4	2	2	4	ClinicalTrials.gov
KEYNOTE‐010	Pembrolizumab	Pembrolizumab	Chemotherapy	339	343	309	8	9	2	8	9	2	ClinicalTrials.gov
KEYNOTE‐021	Pembrolizumab plus chemotherapy	NA	Chemotherapy	59	NA	62	4	NA	0	1	NA	0	ClinicalTrials.gov
KEYNOTE‐024	Pembrolizumab	NA	Chemotherapy	154	NA	150	7	NA	1	7	NA	1	ClinicalTrials.gov
KEYNOTE‐045	Pembrolizumab	NA	Chemotherapy	266	NA	255	6	NA	0	6	NA	0	ClinicalTrials.gov
KEYNOTE‐054	Pembrolizumab	NA	Placebo	509	NA	502	17	NA	3	4	NA	0	Published article
KEYNOTE‐061	Pembrolizumab	NA	Chemotherapy	294	NA	276	8	NA	0	2	NA	0	Published article
KEYNOTE‐189	Pembrolizumab plus chemotherapy	NA	Chemotherapy	405	NA	202	18	NA	5	11	NA	4	Published article
OAK	Atezolizumab	NA	Chemotherapy	609	NA	578	5	NA	1	5	NA	1	ClinicalTrials.gov
ONO‐4538‐12, ATTRACTION‐2	Nivolumab	NA	Placebo	330	NA	161	1	NA	0	1	NA	0	Published article
PACIFIC study	Durvalumab	NA	Placebo	475	NA	234	161	NA	58	16	NA	6	Published article
POPLAR Study	Atezolizumab	NA	Chemotherapy	142	NA	135	1	NA	0	1	NA	0	ClinicalTrials.gov

NA, not available.

### 3.2 All‐grade (grade 1‐5) pneumonitis

The ORs for pairwise comparisons of all‐grade pneumonitis are shown in Table S4. Compared with chemotherapy, nivolumab, pembrolizumab, nivolumab plus ipilimumab therapy were associated with a statistically significant higher risk of all‐grade pneumonitis (nivolumab vs chemotherapy: OR = 5.49, 95% CI: 2.15‐13.98; pembrolizumab vs chemotherapy: OR = 5.40, 95% CI: 2.39‐12.17; nivolumab plus ipilimumab therapy vs chemotherapy: OR = 7.51, 95% CI: 2.23‐25.22), with moderate quality of evidence respectively.

**Figure 3:**
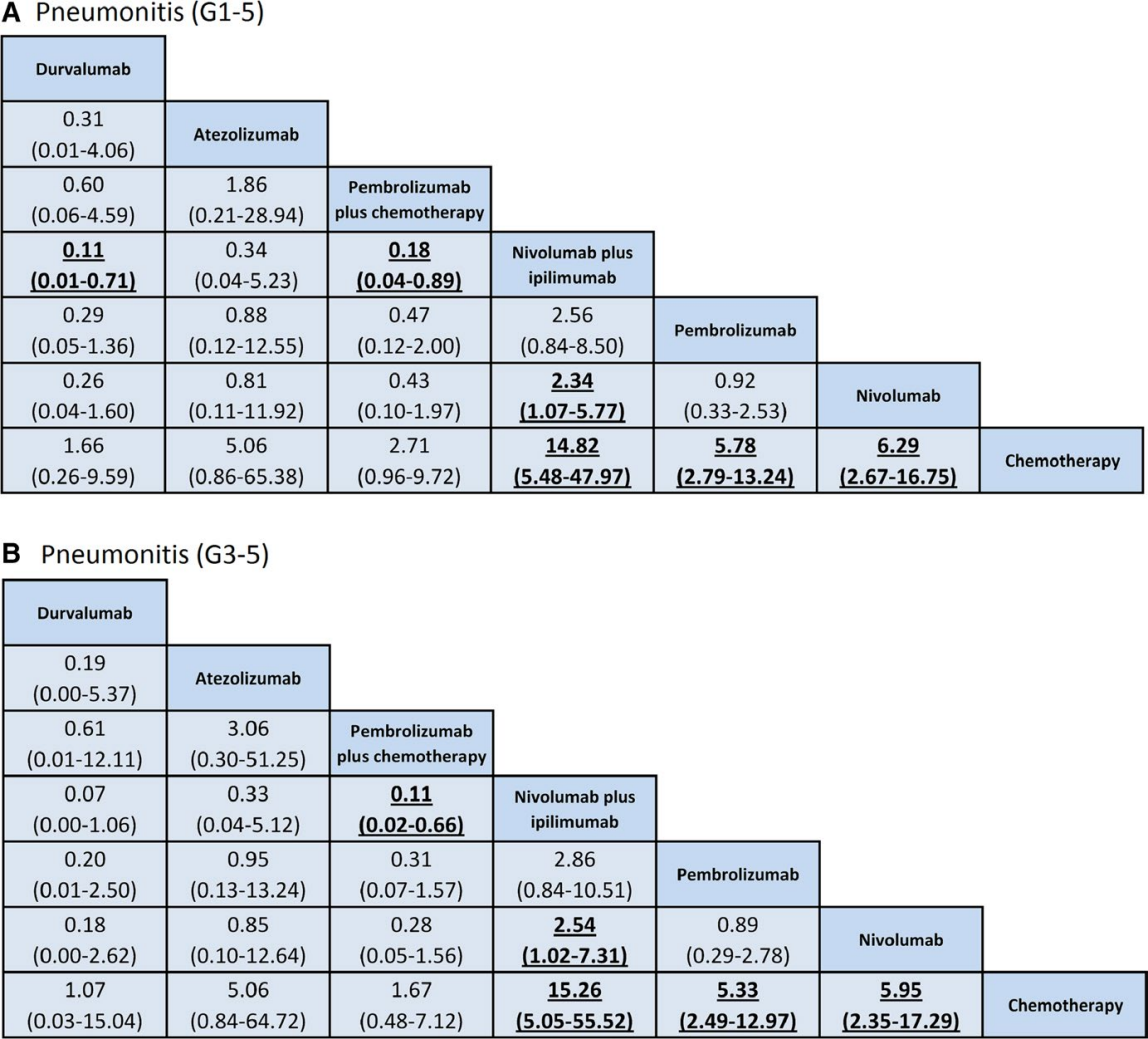
Bayesian network meta‐analysis of pneumonitis. Comparisons should be read from left to right. The column treatment is compared with the row treatment. Bold underline cells are significant. Results represent pooled odds ratios and 95% credible intervals for pneumonitis of Grade 1‐5 (A) and Grade 3‐5 (B). Odds ratio > 1 favors row‐defining treatment

Results of NMA for all‐grade pneumonitis risk were displayed in Figure 3A and Figure S1. Compared with chemotherapy, nivolumab and pembrolizumab were associated with an increased risk of all‐grade pneumonitis (nivolumab vs chemotherapy: OR = 6.29, 95% CrI: 2.67‐16.75; pembrolizumab vs chemotherapy: OR = 5.78, 95% CrI: 2.79‐13.24), with moderate quality of evidence respectively. Compared with chemotherapy, nivolumab plus ipilimumab therapy was associated with an increased risk of all‐grade pneumonitis (OR = 14.82, 95% CrI: 5.48‐47.97), with low quality of evidence. Compared with nivolumab, nivolumab plus ipilimumab therapy was also associated with an increased risk of all‐grade pneumonitis (OR = 2.34, 95% CrI: 1.07‐5.77), with high quality of evidence. Compared with nivolumab plus ipilimumab therapy, pembrolizumab plus chemotherapy was associated with a decreased risk of all‐grade pneumonitis (OR = 0.18, 95% CrI: 0.04‐0.89), with moderate quality of evidence.

### 3.3 High‐grade (grade 3‐5) pneumonitis

The ORs for pairwise comparisons of high‐grade pneumonitis are shown in Table S5. Compared with chemotherapy, nivolumab, pembrolizumab, nivolumab plus ipilimumab therapy were associated with a statistically significant higher risk of high‐grade pneumonitis (nivolumab vs chemotherapy: OR = 5.04, 95% CI: 1.80‐14.15; pembrolizumab vs chemotherapy: OR = 4.88, 95% CI: 2.16‐11.05; nivolumab plus ipilimumab therapy vs chemotherapy: OR = 6.56, 95% CI: 1.47‐29.19), with moderate quality of evidence respectively.

Results of NMA for high‐grade pneumonitis risk were displayed in Figure 3B and Figure S2. Compared with chemotherapy, nivolumab and pembrolizumab were associated with an increased risk of high‐grade pneumonitis (nivolumab vs chemotherapy: OR = 5.95, 95% CrI: 2.35‐17.29; pembrolizumab vs chemotherapy: OR = 5.33, 95% CrI: 2.49‐12.97), with moderate quality of evidence respectively. Nivolumab plus ipilimumab therapy was associated with an increased risk of high‐grade pneumonitis (OR = 15.26, 95% CrI: 5.05‐55.52), with low quality of evidence. Compared with nivolumab, nivolumab plus ipilimumab therapy was associated with an increased risk of high‐grade pneumonitis (OR = 2.54, 95% CrI: 1.02‐7.31), with high quality of evidence. Compared with nivolumab plus ipilimumab therapy, pembrolizumab plus chemotherapy was associated with a decreased risk of high‐grade pneumonitis (OR = 0.11, 95% CrI: 0.02‐0.66), with moderate quality of evidence.

### 3.4 Ranks and meta‐regression analyses

The ranks of all treatments were presented in Table S6. Nivolumab plus ipilimumab therapy had the highest risk of both all‐grade and high‐grade pneumonitis among PD1/PD‐L1 inhibitor‐related therapeutic regimens. Meta‐regression analyses did not reveal any prespecified factors that influenced the estimated effects significantly (Table S7).

### 3.5 Model fit and inconsistence check

The model fit was evaluated using the posterior mean of the residual deviance, which was 42 and 38 for all‐grade and high‐grade pneumonitis, respectively. The model's overall fit was relatively satisfactory. According to the forest plots, the statistically inconsistency between direct and indirect comparisons was low for all‐grade and high‐grade pneumonitis outcomes. All loops were consistent (Figure S3).

### 3.6 Reporting bias and sensitivity analysis

Figure S4 present the adjusted funnel plot for the pneumonitis network. The funnel plots of all‐grade and high‐grade pneumonitis outcomes did not show asymmetry, suggesting no potential risk of reporting bias. Sensitivity analyses based on published data did not indicate any major influence on the outcomes (Figure S5‐S7).

## 4 DISCUSSION

### 4.1 Summary of key findings

This study has 3 key findings: First, there was moderate quality of evidence that nivolumab, pembrolizumab and nivolumab plus ipilimumab therapy increased the risk of all‐grade and high‐grade immune‐related pneumonitis, compared with chemotherapy. Second, nivolumab plus ipilimumab therapy was associated with an increased risk of all‐grade pneumonitis compared with nivolumab, with high quality of evidence. Third, nivolumab plus ipilimumab therapy had the highest risk of both all‐grade and high‐grade pneumonitis among different types of PD1/PD‐L1 inhibitor‐related therapeutic regimens.

### 4.2 Comparison with other studies

Previous published systematic reviews and meta‐analyses regarding the immune‐related risk of pneumonitis have shown that PD1 inhibitors are associated with an increased risk of immune‐related pneumonitis compared with chemotherapy.^8^, ^15^, ^24^ The Bayesian network meta‐analysis in our study allows us to compare the therapeutic regimens indirectly when no head‐to‐head trial existed. There was high to moderate quality evidence showing that nivolumab plus ipilimumab therapy was associated with an increased risk of pneumonitis, compared with chemotherapy and nivolumab respectively.

Two studies have previously investigated the differences in the toxicities of PD1 and PD‐L1 inhibitors.^24^, ^25^ Khunger et al reported a higher incidence of immune‐related pneumonitis with use of PD‐1 inhibitors compared with PD‐L1 inhibitors in patients with nonsmall cell lung cancer.^24^ The summary of incidence of all‐grade and high‐grade pneumonitis was also reported in Table S8 of our study. Pillai et al found a slight increase in pneumonitis risk with PD‐1 inhibitors.^25^ It should be noted that no RCT to date has directly compared the risk of immune‐related pneumonitis between PD‐1 and PD‐L1 inhibitors. We still lack direct evidence to make such conclusion. In our study, indirect comparison showed an increased but not statistically significant risk of immune‐related pneumonitis with use of nivolumab or pembrolizumab compared with durvalumab or atezolizumab, respectively.

### 4.3 Strength and limitations of study

To our knowledge, this is the first systematic review and NMA which provides the most current and structured evidence of immune‐related pneumonitis for PD1/PD‐L1 inhibitor‐related therapeutic regimens. Studies from ClinicalTrials.gov were searched carefully. Both published and unpublished data were extracted. We believe that all relevant RCTs were included in analyses and the publication bias was reduced as much as possible. The main limitation of this study is that indirect comparisons from NMA are very likely to suffer bias through confounding by study‐level characteristics. The results from indirect comparisons should be interpreted with caution as direct evidence is lacking. However, the trial populations and study characteristics were very comparable to the target population of this NMA. We further evaluated the potential confounding factors with meta‐regression analyses, which showed no major influence to our primary results. Secondly, compared with chemotherapy, we did not find significant increase in pneumonitis risk for atezolizumab or durvalumab. It did not indicate that there was no risk of pneumonitis with the use of these 2 agents. Compared with chemotherapy, the direction of risks for durvalumab and atezolizumab were both positive (more than one), despite of lacking significance. One of the potential reasons of lacking significance may due to limited number of trials with positive results for these 2 agents. More RCTs are needed in the future to detect their potential risks further.

### 4.4 Research and clinical implications

Traditionally, most assessments of safety of PD1/PD‐L1 inhibitors come from comparisons with chemotherapy. Despite our current systematic review and NMA provides insight in these comparisons from indirect comparisons, evidence on head‐to‐head comparisons among different PD1/PD‐L1 inhibitor‐related therapeutic regimens is still lacking. New trials comparing between different PD1/PD‐L1 inhibitor‐related therapeutic regimens are needed. Future trials could also be conducted to assess the safety of the combination of PD1/PD‐L1 inhibitor and chemotherapy to enrich the evidence.

Two clinical implications should be noted. First, nivolumab plus ipilimumab therapy had the highest pneumonitis risk among different PD1/PD‐L1 inhibitor‐related therapeutic regimens. The results of both all‐grade and high‐grade pneumonitis outcomes were stable in sensitivity analysis. It should be noted that ipilimumab blocks cytotoxic T‐lymphocyte antigen‐4 (CTLA‐4) as well as augments T‐cell immune response as an immunomodulator. CTLA‐4 has both cell intrinsic activities and cell extrinsic activities. In contrast, immunoregulation by PD‐1 is antigen specific and cell intrinsic.^26^ Consistent with their mechanism of action, immune‐related adverse event rates are more likely to be higher for the combination use of PD1 and CTLA4 inhibitors, compared with using PD1 inhibitors alone. Moreover, immune‐related all‐grade and high‐grade pneumonitis were also significant for both nivolumab and pembrolizumab therapy. Physicians may need to consider the increased pneumonitis risk when choosing these therapies for cancer patients. Second, the combination of chemotherapy and PD1/PD‐L1 inhibitor had a decreased risk of immune‐related pneumonitis, compared with using nivolumab plus ipilimumab. These results may be taken into account by the physicians in decision making when choosing among the different combinations of therapies.

## 5 CONCLUSIONS

This systematic review and network meta‐analysis offers substantial evidence and demonstrates that PD‐1 inhibitor is very likely to result in a higher risk of immune‐related pneumonitis compared with chemotherapy. Nivolumab plus ipilimumab therapy had the highest pneumonitis risk. These findings may be taken into account by the physicians in decision making when choosing among different PD1/PD‐L1 inhibitor‐related therapeutic regimens for cancer patients.

## CONFLICT OF INTEREST

None.

## AUTHOR CONTRIBUTIONS

Yafang Huang: Conceptualization, methodology, investigation, data curation, analysis, resources, writing‐original draft, visualization, supervision, and project administration. **Haiyu Fan:** Investigation, data curation, analysis, writing‐review and editing, and visualization. **Ning Li:** Investigation, data curation, analysis, writing‐review and editing, and visualization. **Juan Du:** Methodology, investigation, writing‐original draft, visualization, and supervision.
