# Supplementary material for: Risk of immune‐related pneumonitis for PD1/PD‐L1 inhibitors: Systematic review and network meta‐analysis
Source: Cancer Med. 2019 Apr 5;8(5):2664–74. doi: 10.1002/cam4.2104 (PMC6536966; doi:10.1002/cam4.2104)

**Online-Only Figures**

**Supplement figure 1 Forest plot of all-grade pneumonitis**

**Supplement figure 2 Forest plot of high-grade pneumonitis**

**Supplement figure 3 Inconsistency plot for the pneumonitis network**

**Supplement figure 4 Comparison – adjusted funnel plot for the pneumonitis network**

**Supplement figure 5 Bayesian network meta-analysis of pneumonitis (sensitivity analysis)**

**Supplement figure 6 Forest plot of all-grade pneumonitis (sensitivity analysis)**

**Supplement figure 7 Forest plot of high-grade pneumonitis (sensitivity analysis)**

**Supplement figure 1 Forest plot of all-grade pneumonitis**


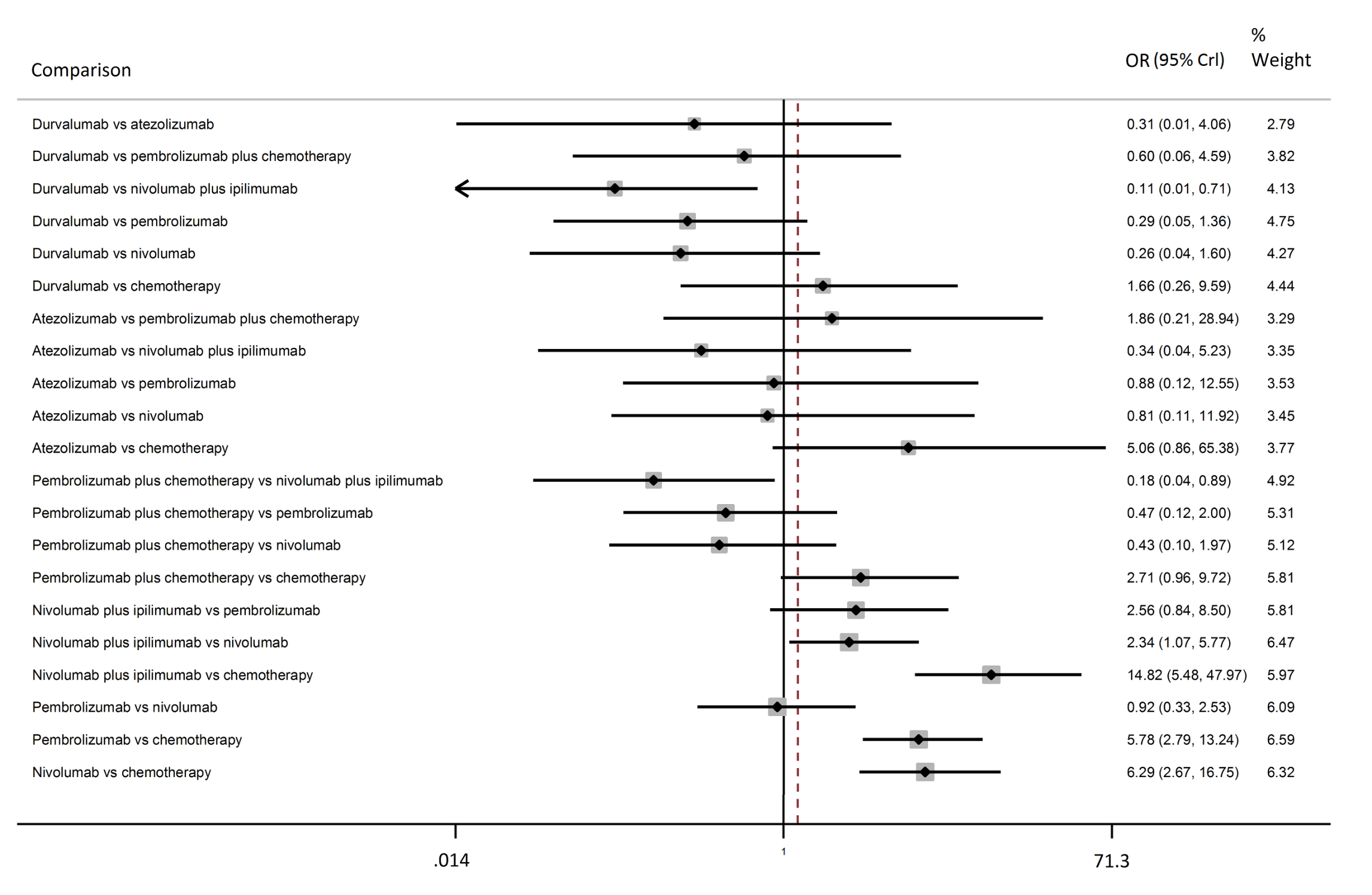


**Supplement figure 2 Forest plot of high-grade pneumonitis**

**
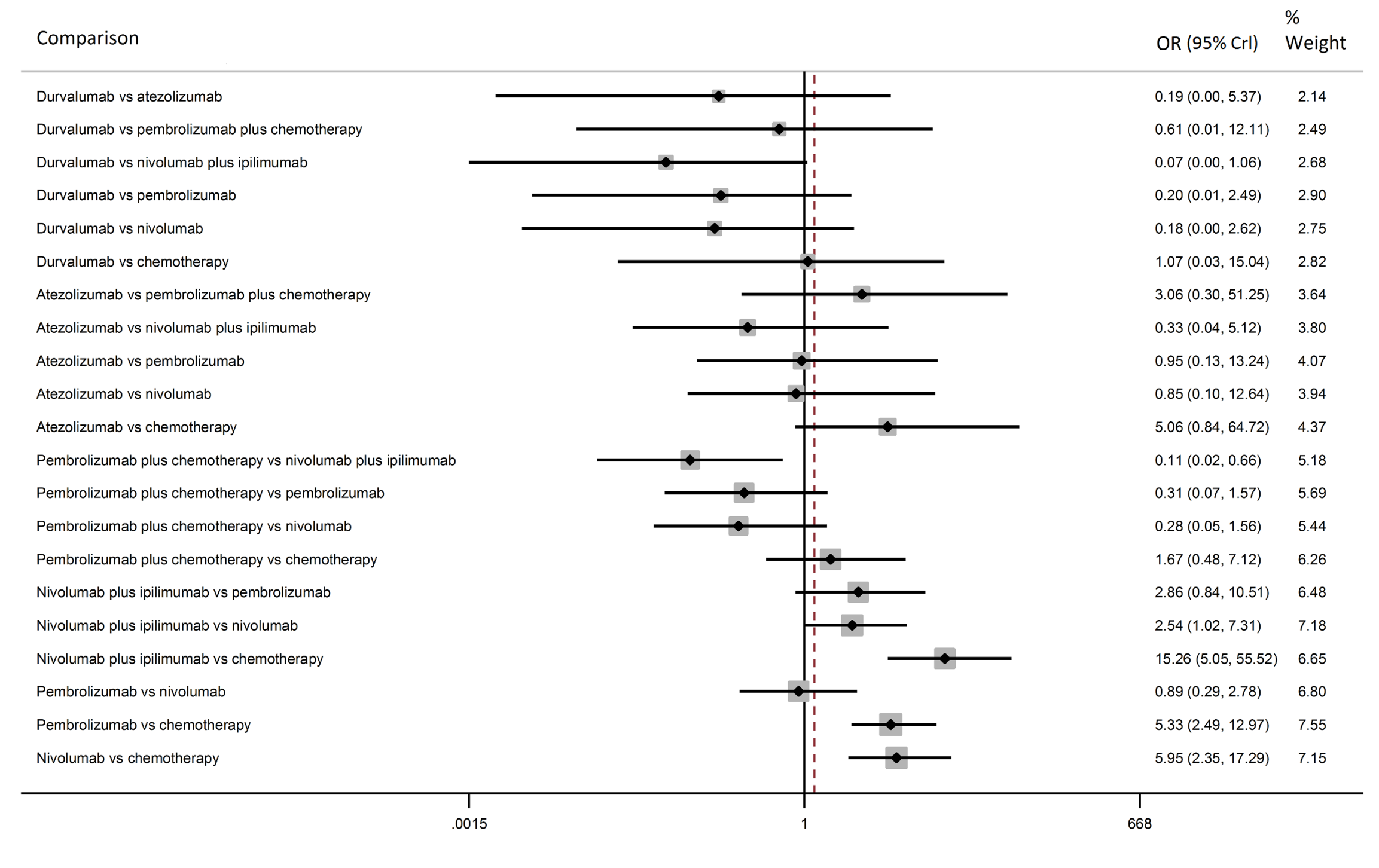
**

**Supplement figure 3 Inconsistency plot for the pneumonitis network**

**
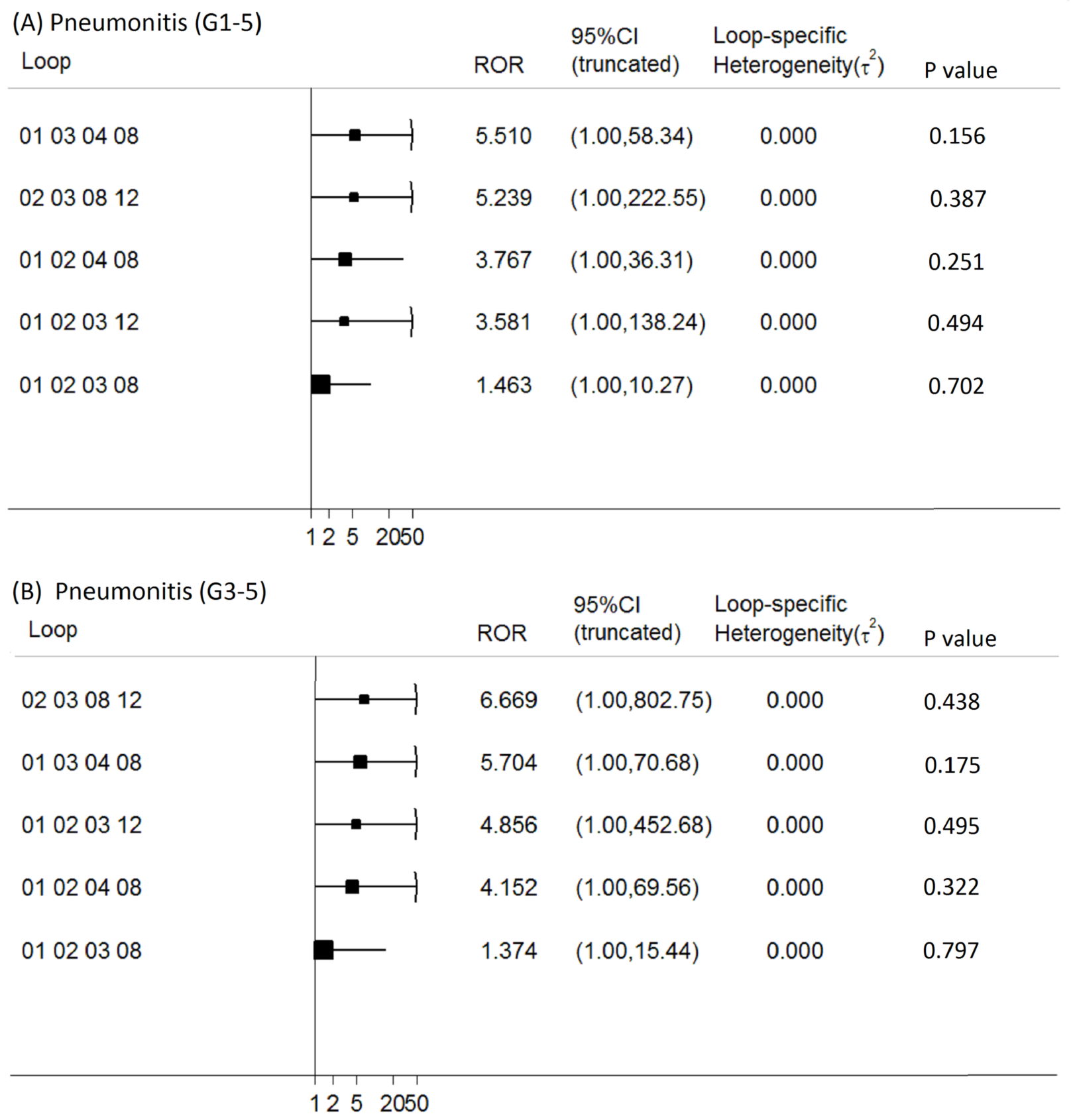
**

01=Chemotherapy; 02=Nivolumab; 03=Pembrolizumab; 04=Nivolumab plus ipilimumab; 08=Ipilimumab; 12=Placebo.

**Supplement figure 4 Comparison – adjusted funnel plot for the pneumonitis network**

**
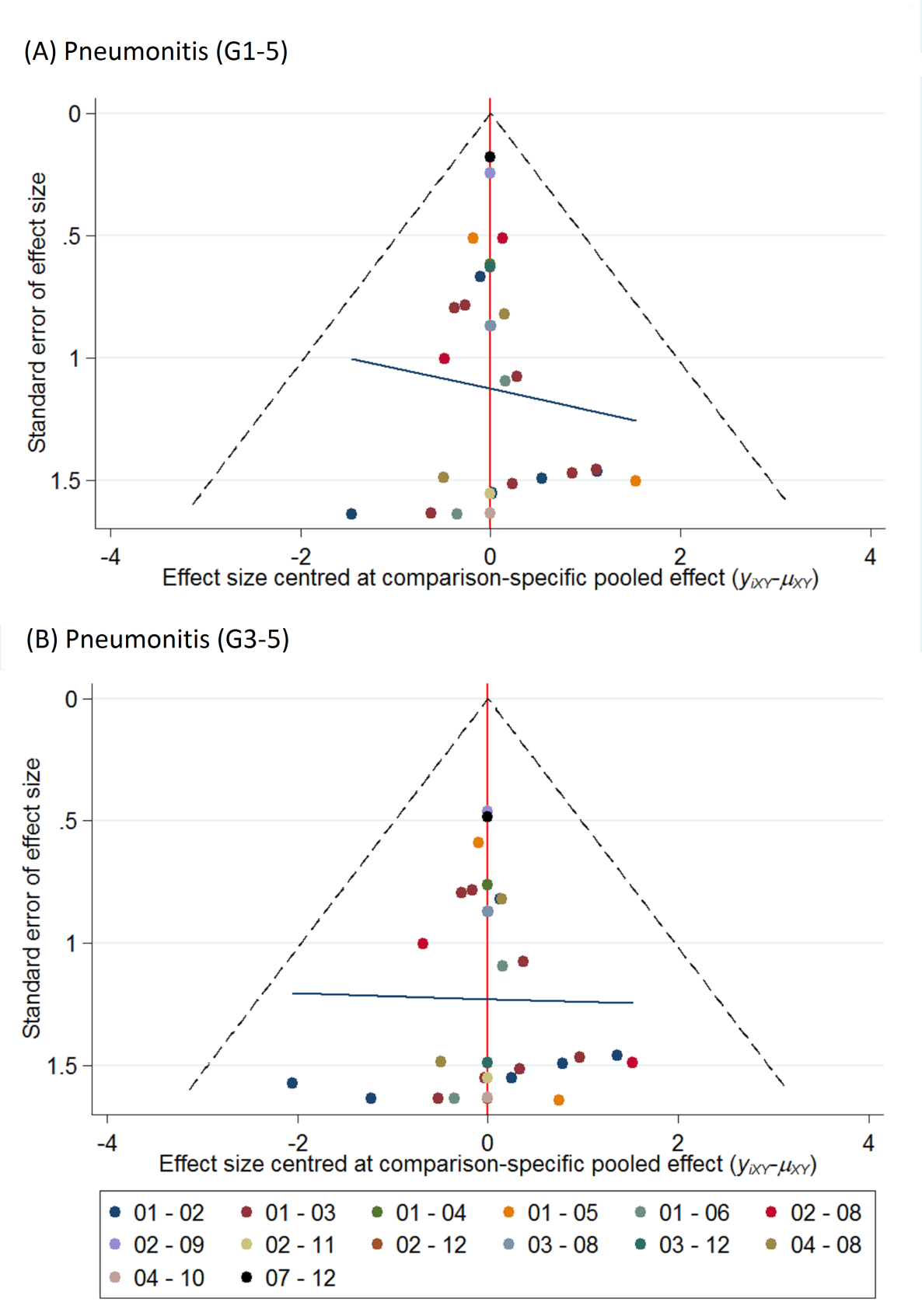
**

The red line represents the null hypothesis that the study-specific effect sizes do not differ from the respective comparison-specific pooled effect estimates. The blue line is the regression line. Different colors correspond to different comparisons. 01=Chemotherapy; 02=Nivolumab; 03=Pembrolizumab; 04=Nivolumab plus ipilimumab; 05=Pembrolizumab plus chemotherapy; 06=Atezolizumab; 07=Durvalumab; 08=Ipilimumab; 09=Everolimus; 10=Sunitinib; 11=Standard therapy; 12=Placebo.

**Supplement figure 5 Bayesian network meta-analysis of pneumonitis (sensitivity analysis)**


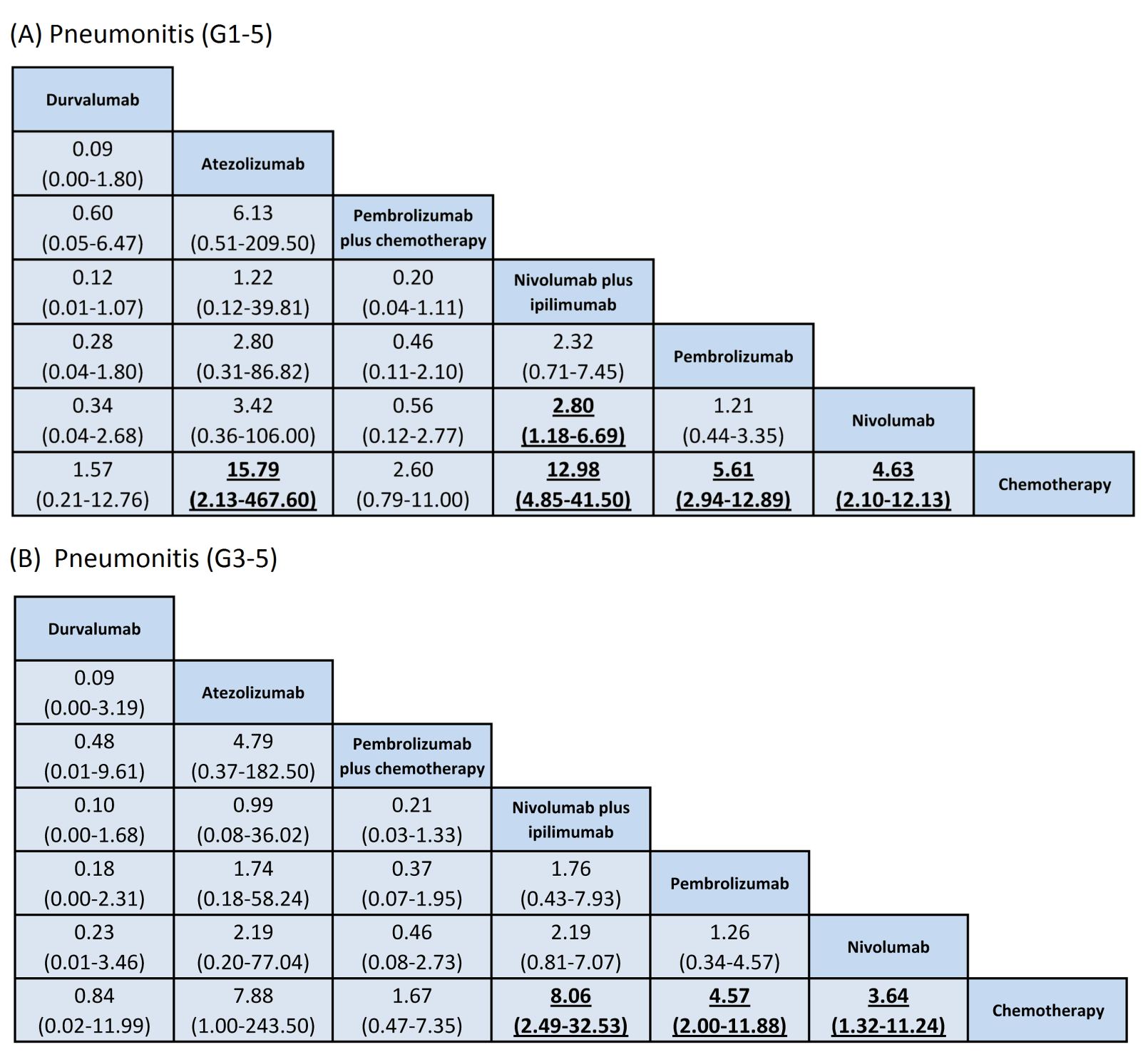


Comparisons should be read from left to right. The column treatment is compared with the row treatment. Bold underline cells are significant. Results represent pooled odds ratios and 95% credible intervals for pneumonitis of Grade 1-5 (A) and Grade 3-5 (B). Odds ratio >1 favors row-defining treatment.

**Supplement figure 6 Forest plot of all-grade pneumonitis (sensitivity analysis)**

**
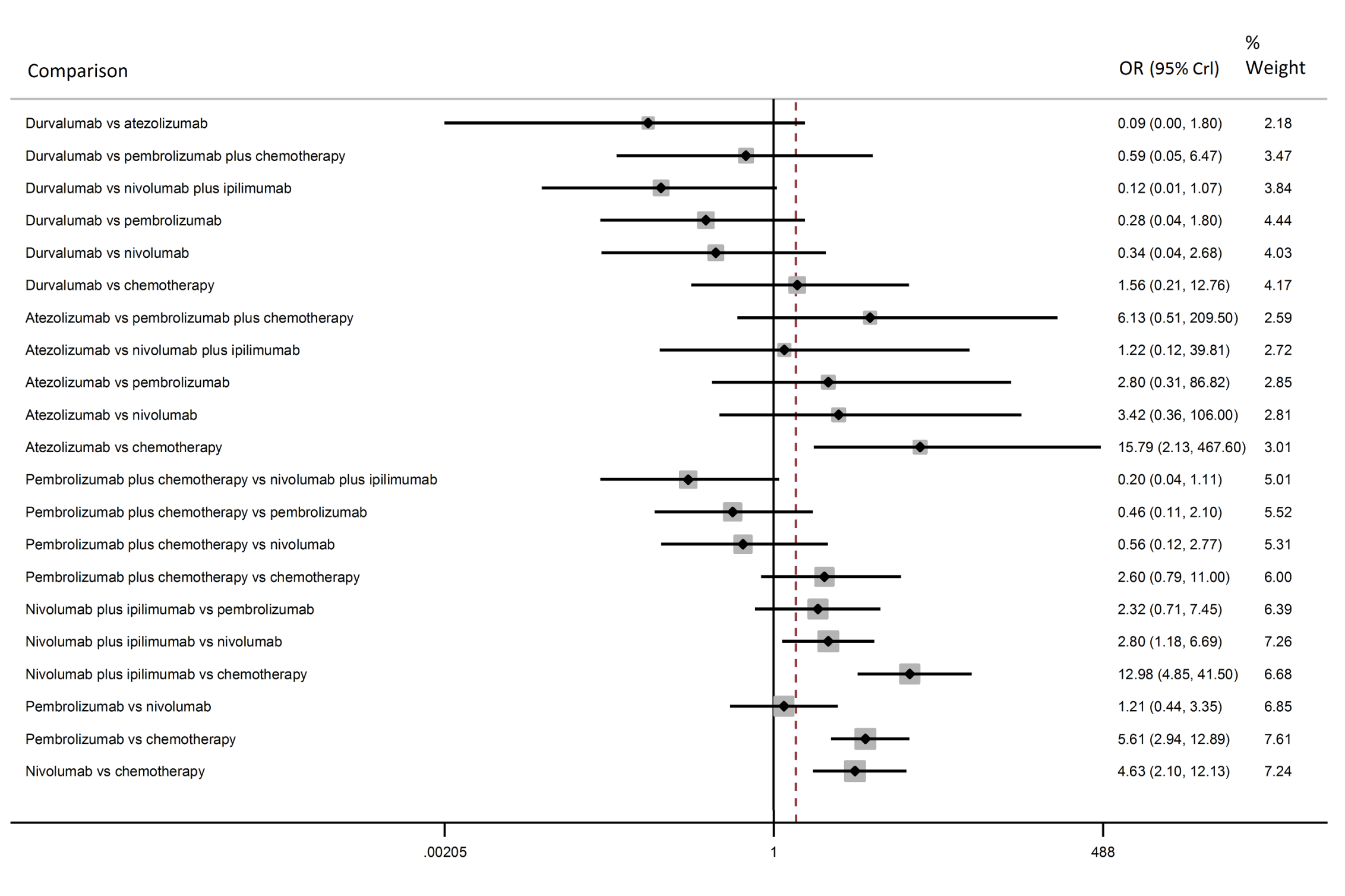
**

**Supplement figure 7 Forest plot of high-grade pneumonitis (sensitivity analysis)**


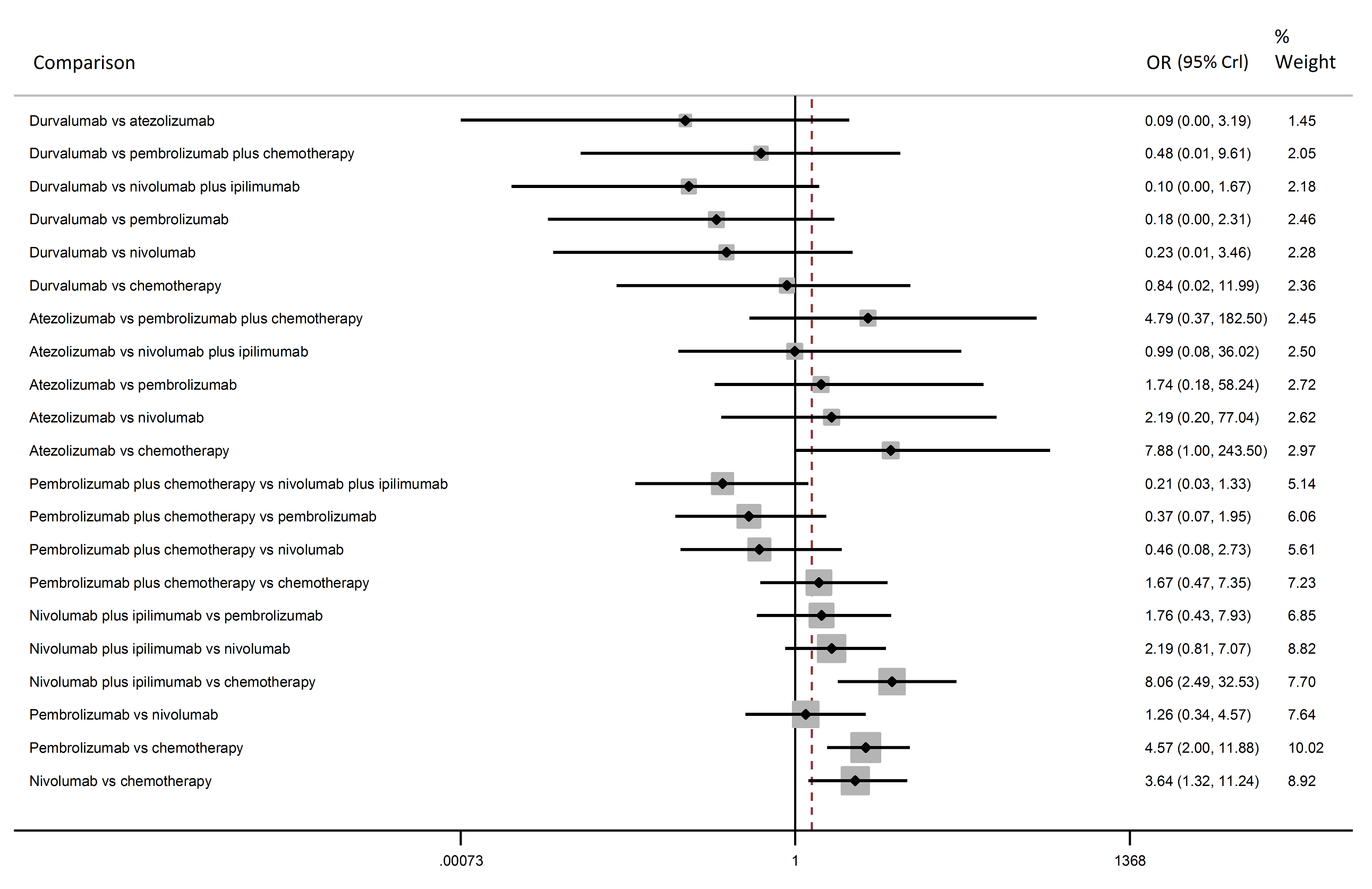

Supplement: Supplementary file 1 [file CAM4-8-2664-s001.docx]
