# Supplementary material for: Risk of immune‐related pneumonitis for PD1/PD‐L1 inhibitors: Systematic review and network meta‐analysis
Source: Cancer Med. 2019 Apr 5;8(5):2664–74. doi: 10.1002/cam4.2104 (PMC6536966; doi:10.1002/cam4.2104)
Supplement: Supplementary file 2 [file CAM4-8-2664-s002.docx]

**Online-Only Tables**

**Supplement table 1 Reference of included articles**

**Supplement table 2 Number of patients with immune-related pneumonitis (data for sensitivity analysis)**

**Supplement table 3 Risk of bias of included studies**

**Supplement table 4 Evaluation of the quality of evidence using GRADE framework for pneumonitis Grade 1-5**

**Supplement table 5 Evaluation of the quality of evidence using GRADE framework for pneumonitis Grade 3-5**

**Supplement table 6 Rank of immune-related pneumonitis among different therapeutic regimens**

**Supplement table 7 Meta-regression for pneumonitis**

**Supplement table 8 Summary of incidence of all-grade and high-grade pneumonitis**

**Supplement table 1 Reference of included articles**

| No. | Trial name | References |
| --- | --- | --- |
| 1 | PACIFIC study | Antonia SJ, Villegas A, Daniel D, et al. Durvalumab after chemoradiotherapy in stage III non-small-cell lung cancer. *N Engl J Med.* 2017;377(20):1919-1929. |
| 2 | KEYNOTE-045 | Bellmunt J, de Wit R, Vaughn DJ, et al. Pembrolizumab as second-line therapy for advanced urothelial carcinoma. *N Engl J Med.* 2017;376(11):1015-1026. |
| 3 | CheckMate 057 | Borghaei H, Paz-Ares L, Horn L, et al. Nivolumab versus docetaxel in advanced nonsquamous non-small-cell lung cancer. *N Engl J Med.* 2015;373(17):1627-1639. |
| 4 | CheckMate 017 | Brahmer J, Reckamp KL, Baas P, et al. Nivolumab versus docetaxel in advanced squamous-cell non-small-cell lung cancer. *N Engl J Med.* 2015;373(2):123-135. |
| 5 | CheckMate 026 | Carbone DP, Reck M, Paz-Ares L, et al. First-line nivolumab in stage IV or recurrent non-small-cell lung cancer. *N Engl J Med.* 2017;376(25):2415-2426. |
| 6 | KEYNOTE-054 | Eggermont AMM, Blank CU, Mandala M, et al. Adjuvant pembrolizumab versus placebo in resected stage III melanoma. *N Engl J Med.* 2018;378(19):1789-1801. |
| 7 | POPLAR Study | Fehrenbacher L, Spira A, Ballinger M, et al. Atezolizumab versus docetaxel for patients with previously treated non-small-cell lung cancer (POPLAR): a multicentre, open-label, phase 2 randomised controlled trial. *Lancet.* 2016;387(10030):1837-1846. |
| 8 | CheckMate 141 | Ferris RL, Blumenschein G, Jr., Fayette J, et al. Nivolumab for recurrent squamous-cell carcinoma of the head and neck. N Engl J Med. 2016;375(19):1856-1867.  Gillison ML, Blumenschein G, Jr., Fayette J, et al. CheckMate 141: 1-year update and subgroup analysis of nivolumab as first-line therapy in patients with recurrent/metastatic head and neck cancer. Oncologist. 2018;(in press). |
| 9 | KEYNOTE-189 | Gandhi L, Rodriguez-Abreu D, Gadgeel S, et al. Pembrolizumab plus chemotherapy in metastatic non-small-cell lung cancer. *N Engl J Med.* 2018;378(22):2078-2092. |
| 10 | CheckMate 227 | Hellmann MD, Ciuleanu TE, Pluzanski A, et al. Nivolumab plus ipilimumab in lung cancer with a high tumor mutational burden. *N Engl J Med.* 2018;378(22):2093-2104. |
| 11 | KEYNOTE-010 | Herbst RS, Baas P, Kim DW, et al. Pembrolizumab versus docetaxel for previously treated, PD-L1-positive, advanced non-small-cell lung cancer (KEYNOTE-010): a randomised controlled trial. *Lancet.* 2016;387(10027):1540-1550. |
| 12 | ONO-4538-12, ATTRACTION-2 | Kang YK, Boku N, Satoh T, et al. Nivolumab in patients with advanced gastric or gastro-oesophageal junction cancer refractory to, or intolerant of, at least two previous chemotherapy regimens (ONO-4538-12, ATTRACTION-2): a randomised, double-blind, placebo-controlled, phase 3 trial. *Lancet.* 2017;390(10111):2461-2471. |
| 13 | KEYNOTE-021 | Langer CJ, Gadgeel SM, Borghaei H, et al. Carboplatin and pemetrexed with or without pembrolizumab for advanced, non-squamous non-small-cell lung cancer: a randomised, phase 2 cohort of the open-label KEYNOTE-021 study. *Lancet Oncol.* 2016;17(11):1497-1508. |
| 14 | CheckMate 037 | Larkin J, Minor D, D'Angelo S, et al. Overall survival in patients with advanced melanoma who received nivolumab versus investigator's choice chemotherapy in CheckMate 037: a randomized, controlled, open-label phase III trial. J Clin Oncol. 2018;36(4):383-390.  Weber JS, D'Angelo SP, Minor D, et al. Nivolumab versus chemotherapy in patients with advanced melanoma who progressed after anti-CTLA-4 treatment (CheckMate 037): a randomised, controlled, open-label, phase 3 trial. Lancet Oncol. 2015;16(4):375-384. |
| 15 | CheckMate 067 | Larkin J, Chiarion-Sileni V, Gonzalez R, et al. Combined nivolumab and ipilimumab or monotherapy in untreated melanoma. N Engl J Med. 2015;373(1):23-34.  Wolchok JD, Chiarion-Sileni V, Gonzalez R, et al. Overall survival with combined nivolumab and ipilimumab in advanced melanoma. N Engl J Med. 2017;377(14):1345-1356. |
| 16 | CheckMate 214 | Motzer RJ, Tannir NM, McDermott DF, et al. Nivolumab plus ipilimumab versus sunitinib in advanced renal-cell carcinoma. *N Engl J Med.* 2018;378(14):1277-1290. |
| 17 | CheckMate 025 | Motzer RJ, Escudier B, McDermott DF, et al. Nivolumab versus everolimus in advanced renal-cell carcinoma. *N Engl J Med.* 2015;373(19):1803-1813. |
| 18 | CheckMate 069 | Postow MA, Chesney J, Pavlick AC, et al. Nivolumab and ipilimumab versus ipilimumab in untreated melanoma. *N Engl J Med.* 2015;372(21):2006-2017. |
| 19 | KEYNOTE-024 | Reck M, Rodriguez-Abreu D, Robinson AG, et al. Pembrolizumab versus chemotherapy for PD-L1-positive non-small-cell lung cancer. *N Engl J Med.* 2016;375(19):1823-1833. |
| 20 | KEYNOTE-002 | Ribas A, Puzanov I, Dummer R, et al. Pembrolizumab versus investigator-choice chemotherapy for ipilimumab-refractory melanoma (KEYNOTE-002): a randomised, controlled, phase 2 trial. Lancet Oncol. 2015;16(8):908-918.  Hamid O, Puzanov I, Dummer R, et al. Final analysis of a randomised trial comparing pembrolizumab versus investigator-choice chemotherapy for ipilimumab-refractory advanced melanoma. Eur J Cancer. 2017;86:37-45. |
| 21 | OAK | Rittmeyer A, Barlesi F, Waterkamp D, et al. Atezolizumab versus docetaxel in patients with previously treated non-small-cell lung cancer (OAK): a phase 3, open-label, multicentre randomised controlled trial. *Lancet.* 2017;389(10066):255-265. |
| 22 | CheckMate 066 | Robert C, Long GV, Brady B, et al. Nivolumab in previously untreated melanoma without BRAF mutation. *N Engl J Med.* 2015;372(4):320-330. |
| 23 | KEYNOTE-006 | Robert C, Schachter J, Long GV, et al. Pembrolizumab versus ipilimumab in advanced melanoma. N Engl J Med. 2015;372(26):2521-2532.  Schachter J, Ribas A, Long GV, et al. Pembrolizumab versus ipilimumab for advanced melanoma: final overall survival results of a multicentre, randomised, open-label phase 3 study (KEYNOTE-006). Lancet. 2017;390(10105):1853-1862. |
| 24 | KEYNOTE-061 | Shitara K, Ozguroglu M, Bang YJ, et al. Pembrolizumab versus paclitaxel for previously treated, advanced gastric or gastro-oesophageal junction cancer (KEYNOTE-061): a randomised, open-label, controlled, phase 3 trial. *Lancet.* 2018;(in press). |
| 25 | CheckMate 238 | Weber J, Mandala M, Del Vecchio M, et al. Adjuvant nivolumab versus ipilimumab in resected stage III or IV melanoma. *N Engl J Med.* 2017;377(19):1824-1835. |

**Supplement table 2 Number of patients with immune-related pneumonitis (data for sensitivity analysis)**

| Trial name | Types of treatment | | | Number of patients for adverse events | | | Pneumonitis events  (Grade 1 -5) | | | Pneumonitis events  (Grade 3-5) | | |
| --- | --- | --- | --- | --- | --- | --- | --- | --- | --- | --- | --- | --- |
|  | Arm 1 | Arm 2 | Arm 3 | Arm 1 | Arm 2 | Arm 3 | Arm 1 | Arm 2 | Arm 3 | Arm 1 | Arm 2 | Arm 3 |
| CheckMate 017 | Nivolumab | NA | Chemotherapy | 131 | NA | 129 | 6 | NA | 0 | 1 | NA | 0 |
| CheckMate 025 | Nivolumab | NA | Everolimus | 406 | NA | 397 | 16 | NA | 58 | 6 | NA | 11 |
| CheckMate 026 | Nivolumab | NA | Chemotherapy | 267 | NA | 263 | 7 | NA | 1 | 4 | NA | 0 |
| CheckMate 037 | Nivolumab | NA | Chemotherapy | 268 | NA | 102 | 7 | NA | 0 | 0 | NA | 0 |
| CheckMate 057 | Nivolumab | NA | Chemotherapy | 287 | NA | 268 | 4 | NA | 0 | 3 | NA | 0 |
| CheckMate 066 | Nivolumab | NA | Chemotherapy | 206 | NA | 205 | 3 | NA | 0 | 0 | NA | 0 |
| CheckMate 067 | Nivolumab | Nivolumab plus ipilimumab | Ipilimumab | 313 | 313 | 311 | 4 | 20 | 5 | 1 | 3 | 1 |
| CheckMate 069 | Nivolumab plus ipilimumab | NA | Ipilimumab | 94 | NA | 46 | 10 | NA | 2 | 2 | NA | 1 |
| CheckMate 141 | Nivolumab | NA | Standard therapy | 236 | NA | 111 | 5 | NA | 1 | 2 | NA | 0 |
| CheckMate 214 | Nivolumab plus ipilimumab | NA | Sunitinib | 547 | NA | 535 | 1 | NA | 0 | 1 | NA | 0 |
| CheckMate 227 | Nivolumab plus ipilimumab | Nivolumab | Chemotherapy | 576 | 391 | 570 | 22 | 9 | 3 | 13 | 6 | 2 |
| CheckMate 238 | Nivolumab | NA | Ipilimumab | 452 | NA | 453 | 6 | NA | 11 | 0 | NA | 4 |
| KEYNOTE-002 | Pembrolizumab | Pembrolizumab | Chemotherapy | 178 | 179 | 171 | 3 | 3 | 0 | 0 | 2 | 0 |
| KEYNOTE-006 | Pembrolizumab | Pembrolizumab | Ipilimumab | 278 | 277 | 256 | 1 | 5 | 1 | 0 | 1 | 1 |
| KEYNOTE-010 | pembrolizumab | Pembrolizumab | Chemotherapy | 339 | 343 | 309 | 16 | 15 | 6 | 7 | 7 | 2 |
| KEYNOTE-021 | Pembrolizumab plus chemotherapy | NA | Chemotherapy | 59 | NA | 62 | 3 | NA | 0 | 1 | NA | 0 |
| KEYNOTE-024 | Pembrolizumab | NA | Chemotherapy | 154 | NA | 150 | 9 | NA | 1 | 4 | NA | 1 |
| KEYNOTE-045 | Pembrolizumab | NA | Chemotherapy | 266 | NA | 255 | 11 | NA | 1 | 6 | NA | 0 |
| KEYNOTE-054 | Pembrolizumab | NA | Placebo | 509 | NA | 502 | 17 | NA | 3 | 4 | NA | 0 |
| KEYNOTE-061 | Pembrolizumab | NA | Chemotherapy | 294 | NA | 276 | 8 | NA | 0 | 2 | NA | 0 |
| KEYNOTE-189 | Pembrolizumab plus chemotherapy | NA | Chemotherapy | 405 | NA | 202 | 18 | NA | 5 | 11 | NA | 4 |
| OAK | Atezolizumab | NA | Chemotherapy | 609 | NA | 578 | 6 | NA | 0 | 4 | NA | 0 |
| ONO-4538-12, ATTRACTION-2 | Nivolumab | NA | Placebo | 330 | NA | 161 | 1 | NA | 0 | 1 | NA | 0 |
| PACIFIC study | Durvalumab | NA | Placebo | 475 | NA | 234 | 161 | NA | 58 | 16 | NA | 6 |
| POPLAR Study | Atezolizumab | NA | Chemotherapy | 142 | NA | 135 | 4 | NA | 0 | 1 | NA | 0 |

NA = not available

**Supplement table 3 Risk of bias of included studies**

| Trial name | Random sequence generation | Allocation concealment | Blinding of participants and personnel | Blinding of outcome assessment | Incomplete outcome data | Selective reporting |
| --- | --- | --- | --- | --- | --- | --- |
| CheckMate 017 | Low | Low | High | High | High | High |
| CheckMate 025 | Low | Low | High | High | High | High |
| CheckMate 026 | Low | Low | Low | High | High | High |
| CheckMate 037 | Low | Low | Low | High | High | High |
| CheckMate 057 | Low | Low | High | High | High | High |
| CheckMate 066 | Low | Low | Low | Low | High | High |
| CheckMate 067 | Low | Low | Low | Low | High | High |
| CheckMate 069 | Low | Low | Low | Low | High | High |
| CheckMate 141 | Low | Low | High | High | High | High |
| CheckMate 214 | Low | Low | Low | High | Unclear | Unclear |
| CheckMate 227 | Low | Low | Low | High | Unclear | Unclear |
| CheckMate 238 | Low | Low | Low | Low | Unclear | Unclear |
| KEYNOTE-002 | Low | Low | High | High | High | High |
| KEYNOTE-006 | Low | Low | Low | High | High | High |
| KEYNOTE-010 | Low | Low | High | High | High | High |
| KEYNOTE-021 | Low | Low | High | High | High | High |
| KEYNOTE-024 | Low | Unclear | High | High | High | High |
| KEYNOTE-045 | Low | Unclear | High | High | High | High |
| KEYNOTE-054 | Low | Low | Low | Low | Unclear | Unclear |
| KEYNOTE-061 | Low | Low | Low | High | Unclear | Unclear |
| KEYNOTE-189 | Low | Low | Low | Low | Unclear | Unclear |
| OAK | Low | Low | High | High | High | High |
| ONO-4538-12, ATTRACTION-2 | Low | Low | Low | Low | Unclear | Unclear |
| PACIFIC study | Low | Low | Low | Low | Unclear | Unclear |
| POPLAR Study | Low | Low | High | High | High | High |

**Supplement table 4 Evaluation of the quality of evidence using GRADE framework for pneumonitis Grade 1-5**

| Comparison | | | Direct evidence | | Indirect evidence | | Network meta-analysis | |
| --- | --- | --- | --- | --- | --- | --- | --- | --- |
|  |  |  | Odds ratio (95% confidence interval) | Quality of evidence | Odds ratio (95% credible interval) | Quality of evidence | Odds ratio (95% credible interval) | Quality of evidence |
| Atezolizumab | vs | Chemotherapy | 4.08(0.68-24.35) | Moderate* | Not estimable | Not estimable | 5.06(0.86-65.38) | Low¶,** |
| Atezolizumab | vs | Nivolumab | - | - | 0.81(0.11-11.92) | Moderate** | 0.81(0.11-11.92) | Moderate** |
| Atezolizumab | vs | Pembrolizumab | - | - | 0.88(0.12-12.55) | Moderate** | 0.88(0.12-12.55) | Moderate** |
| Atezolizumab | vs | Nivolumab plus ipilimumab | - | - | 0.34(0.04-5.23) | Moderate** | 0.34(0.04-5.23) | Moderate** |
| Atezolizumab | vs | Pembrolizumab plus chemotherapy | - | - | 1.86(0.21-28.94) | Moderate** | 1.86(0.21-28.94) | Moderate** |
| Durvalumab | vs | Chemotherapy | - | - | 1.66(0.26-9.59) | Moderate** | 1.66(0.26-9.59) | Moderate** |
| Durvalumab | vs | Nivolumab | - | - | 0.26(0.04-1.60) | Moderate** | 0.26(0.04-1.60) | Moderate** |
| Durvalumab | vs | Pembrolizumab | - | - | 0.29(0.05-1.36) | Moderate** | 0.29(0.05-1.36) | Moderate** |
| Durvalumab | vs | Nivolumab plus ipilimumab | - | - | 0.11(0.01-0.71) | Moderate** | 0.11(0.01-0.71) | Moderate** |
| Durvalumab | vs | Pembrolizumab plus chemotherapy | - | - | 0.60(0.06-4.59) | Moderate** | 0.60(0.06-4.59) | Moderate** |
| Durvalumab | vs | Atezolizumab | - | - | 0.31(0.01-4.06) | Moderate** | 0.31(0.01-4.06) | Moderate** |
| Nivolumab | vs | Chemotherapy | 5.49(2.15-13.98) | Moderate* | Not estimable | Not estimable | 6.29(2.67-16.75) | Moderate¶ |
| Nivolumab plus ipilimumab | vs | Chemotherapy | 7.51(2.23-25.22) | Moderate‡ | Not estimable | Not estimable | 14.82(5.48-47.97) | Low¶,** |
| Nivolumab plus ipilimumab | vs | Nivolumab | - | - | 2.34(1.07-5.77) | High | 2.34(1.07-5.77) | High |
| Nivolumab plus ipilimumab | vs | Pembrolizumab | - | - | 2.56(0.84-8.50) | High | 2.56(0.84-8.50) | High |
| Pembrolizumab | vs | Chemotherapy | 5.40(2.39-12.17) | Moderate* | Not estimable | Not estimable | 5.78(2.79-13.24) | Moderate* |
| Pembrolizumab | vs | Nivolumab | - | - | 0.92(0.33-2.53) | High | 0.92(0.33-2.53) | High |
| Pembrolizumab plus chemotherapy | vs | Chemotherapy | 2.44(0.69-8.68) | Moderate* | Not estimable | Not estimable | 2.71(0.96-9.72) | Moderate¶ |
| Pembrolizumab plus chemotherapy | vs | Nivolumab | - | - | 0.43(0.10-1.97) | Moderate** | 0.43(0.10-1.97) | Moderate** |
| Pembrolizumab plus chemotherapy | vs | Pembrolizumab | - | - | 0.47(0.12-2.00) | Moderate** | 0.47(0.12-2.00) | Moderate** |
| Pembrolizumab plus chemotherapy | vs | Nivolumab plus ipilimumab | - | - | 0.18(0.04-0.89) | Moderate** | 0.18(0.04-0.89) | Moderate** |

* Inadequate concealment of allocation or blinding; †Heterogeneity of outcome definition or outcome assessing between each trial; ‡Wide confidence intervals and few events; §Large I^2^; ¶Contributing direct evidence of moderate quality; ** Imprecision.

**Supplement table 5 Evaluation of the quality of evidence using GRADE framework for pneumonitis Grade 3-5**

| Comparison | | | Direct evidence | | Indirect evidence | | Network meta-analysis | |
| --- | --- | --- | --- | --- | --- | --- | --- | --- |
|  |  |  | Odds ratio (95% confidence interval) | Quality of evidence | Odds ratio (95% credible interval) | Quality of evidence | Odds ratio (95% credible interval) | Quality of evidence |
| Atezolizumab | vs | Chemotherapy | 4.08(0.68-24.35) | Moderate* | Not estimable | Not estimable | 5.06(0.84-64.72) | Low¶,** |
| Atezolizumab | vs | Nivolumab | - | - | 0.85(0.10-12.64) | Moderate** | 0.85(0.10-12.64) | Moderate** |
| Atezolizumab | vs | Pembrolizumab | - | - | 0.95(0.13-13.24) | Moderate** | 0.95(0.13-13.24) | Moderate** |
| Atezolizumab | vs | Nivolumab plus ipilimumab | - | - | 0.33(0.04-5.12) | Moderate** | 0.33(0.04-5.12) | Moderate** |
| Atezolizumab | vs | Pembrolizumab plus chemotherapy | - | - | 3.06(0.30-51.25) | Moderate** | 3.06(0.30-51.25) | Moderate** |
| Durvalumab | vs | Chemotherapy | - | - | 1.07(0.03-15.04) | Moderate** | 1.07(0.03-15.04) | Moderate** |
| Durvalumab | vs | Nivolumab | - | - | 0.18(0.00-2.62) | Moderate** | 0.18(0.00-2.62) | Moderate** |
| Durvalumab | vs | Pembrolizumab | - | - | 0.20(0.01-2.50) | Moderate** | 0.20(0.01-2.50) | Moderate** |
| Durvalumab | vs | Nivolumab plus ipilimumab | - | - | 0.07(0.00-1.06) | Moderate** | 0.07(0.00-1.06) | Moderate** |
| Durvalumab | vs | Pembrolizumab plus chemotherapy | - | - | 0.61(0.01-12.11) | Moderate** | 0.61(0.01-12.11) | Moderate** |
| Durvalumab | vs | Atezolizumab | - | - | 0.19(0.00-5.37) | Moderate** | 0.19(0.00-5.37) | Moderate** |
| Nivolumab | vs | Chemotherapy | 5.04(1.80-14.15) | Moderate* | Not estimable | Not estimable | 5.95(2.35-17.29) | Moderate¶ |
| Nivolumab plus ipilimumab | vs | Chemotherapy | 6.56(1.47-29.19) | Moderate‡ | Not estimable | Not estimable | 15.26(5.05-55.52) | Low¶,** |
| Nivolumab plus ipilimumab | vs | Nivolumab | - | - | 2.54(1.02-7.31) | High | 2.54(1.02-7.31) | High |
| Nivolumab plus ipilimumab | vs | Pembrolizumab | - | - | 2.86(0.84-10.51) | High | 2.86(0.84-10.51) | High |
| Pembrolizumab | vs | Chemotherapy | 4.88(2.16-11.05) | Moderate* | Not estimable | Not estimable | 5.33(2.49-12.97) | Moderate* |
| Pembrolizumab | vs | Nivolumab | - | - | 0.89(0.29-2.78) | High | 0.89(0.29-2.78) | High |
| Pembrolizumab plus chemotherapy | vs | Chemotherapy | 1.52(0.51-4.52) | Moderate* | Not estimable | Not estimable | 1.67(0.48-7.12) | Moderate¶ |
| Pembrolizumab plus chemotherapy | vs | Nivolumab | - | - | 0.28(0.05-1.56) | Moderate** | 0.28(0.05-1.56) | Moderate** |
| Pembrolizumab plus chemotherapy | vs | Pembrolizumab | - | - | 0.31(0.07-1.57) | Moderate** | 0.31(0.07-1.57) | Moderate** |
| Pembrolizumab plus chemotherapy | vs | Nivolumab plus ipilimumab | - | - | 0.11(0.02-0.66) | Moderate** | 0.11(0.02-0.66) | Moderate** |

* Inadequate concealment of allocation or blinding; †Heterogeneity of outcome definition or outcome assessing between each trial; ‡Wide confidence intervals and few events; §Large I^2^; ¶Contributing direct evidence of moderate quality; ** Imprecision.

**Supplement table 6 Rank of immune-related pneumonitis risk among different therapeutic regimens**

|  | Rank, Median (95% CrI) | |
| --- | --- | --- |
|  | Pneumonitis (G1-5) | Pneumonitis (G3-5) |
| Main results |  |  |
| Chemotherapy | 11 (8-12) | 10 (8-12) |
| Nivolumab | 5 (3- 8) | 5 (3- 8) |
| Pembrolizumab | 6 (2- 8) | 6 (2- 8) |
| Nivolumab plus ipilimumab | 2 (1- 5) | 2 (1- 5) |
| Pembrolizumab plus chemotherapy | 8 (3-11) | 9 (4-12) |
| Atezolizumab | 6 (1-11) | 6 (1-11) |
| Durvalumab | 9 (4-12) | 10 (2-12) |
| Ipilimumab | 4 (2- 7) | 3 (1- 7) |
| Everolimus | 1 (1- 5) | 3 (1- 8) |
| Sunitinib | 8 (1-12) | 7 (1-12) |
| Standard therapy | 10 (1-12) | 9 (1-12) |
| Placebo | 10 (7-12) | 10 (5-12) |
| Sensitivity analysis |  |  |
| Chemotherapy | 10 (8-12) | 10 (7-12) |
| Nivolumab | 6 (3- 9) | 6 (3- 9) |
| Pembrolizumab | 5 (2- 8) | 5 (2- 8) |
| Nivolumab plus ipilimumab | 3 (1- 5) | 3 (1- 7) |
| Pembrolizumab plus chemotherapy | 8 (3-11) | 8 (3-12) |
| Atezolizumab | 2 (1- 9) | 3 (1-10) |
| Durvalumab | 9 (3-12) | 10 (2-12) |
| Ipilimumab | 6 (3- 9) | 3 (1- 7) |
| Everolimus | 2 (1- 5) | 3 (1- 9) |
| Sunitinib | 8 (1-12) | 8 (1-12) |
| Standard therapy | 9 (3-12) | 10 (1-12) |
| Placebo | 11 (6-12) | 11 (5-12) |

**Supplement table 7 Meta-regression for the outcomes**

| Variable | Regression coefficient, Mean (95% CrI) | |
| --- | --- | --- |
|  | Pneumonitis (G1-5) | Pneumonitis (G3-5) |
| Median age | 0.014(-0.445-0.478) | 0.184(-0.318-0.691) |
| Percentage of male | -0.162(-3.447-3.025) | -0.002(-3.755-3.509) |
| Line of treatment | 0.308(-1.093-1.750) | 0.136(-1.370-1.721) |
| Study phase | 0.082(-2.580-2.075) | 0.004(-2.619-2.009) |
| Whether double-blind was used | -0.992(-3.156-1.087) | -0.378(-2.791-2.285) |

**Supplement table 8 Summary of incidence of all-grade and high-grade pneumonitis**

| Pneumonitis | Therapy (intervention vs control) | Incidence (%) | |
| --- | --- | --- | --- |
|  |  | Intervention | Control |
| All-grade | Nivolumab vs chemotherapy | 1.87 | 0.20 |
|  | Nivolumab vs ipilimumab | 1.05 | 1.70 |
|  | Nivolumab plus ipilimumab vs chemotherapy | 3.82 | 0.53 |
|  | Nivolumab plus ipilimumab vs ipilimumab | 2.70 | 0.56 |
|  | Pembrolizumab vs chemotherapy | 2.40 | 0.30 |
|  | Pembrolizumab vs ipilimumab | 0.72 | 1.56 |
|  | Pembrolizumab plus chemotherapy vs chemotherapy | 4.74 | 1.89 |
|  | Atezolizumab vs chemotherapy | 0.80 | 0.14 |
| High-grade | Nivolumab vs chemotherapy | 1.42 | 0.13 |
|  | Nivolumab vs ipilimumab | 0.26 | 0.79 |
|  | Nivolumab plus ipilimumab vs chemotherapy | 2.26 | 0.35 |
|  | Nivolumab plus ipilimumab vs ipilimumab | 2.70 | 0.56 |
|  | Pembrolizumab vs chemotherapy | 2.05 | 0.30 |
|  | Pembrolizumab vs ipilimumab | 0.72 | 1.56 |
|  | Pembrolizumab plus chemotherapy vs chemotherapy | 2.59 | 1.52 |
|  | Atezolizumab vs chemotherapy | 0.80 | 0.14 |
